# Identification of a De Novoc.1000delA ANK1 mutation associated to hereditary spherocytosis in a neonate with Coombs-negative hemolytic jaundice-case reports and review of the literature 

**DOI:** 10.1186/s12920-021-00912-3

**Published:** 2021-03-11

**Authors:** Lichun Xie, Zhihao Xing, Changgang Li, Si-xi Liu, Fei-qiu Wen

**Affiliations:** 1 The First Affiliated Hospital, Jinan University, Guangzhou, Guangdong China; 2 Department of Hematology/Oncology, Shenzhen Children’s Hospital, No. 7019 Yitian Rd, Shenzhen, Guangdong China

**Keywords:** ANK1 gene, Case report, Frame shift mutation, Hereditary spherocytosis, Neonate

## Abstract

**Background:**

To strengthen the understanding of Hereditary Spherocytosis (HS) and determine the disease-causing mutation present with neonatal jaundice. HS is a hemolytic condition resulting from various erythrocyte membrane defects. Many different mutations result in HS, including mutations in *ANK1*.

**Case presentation:**

A term neonate presented at ten hours with severe jaundice requiring exchange transfusion. At two months he was hospitalized due to repeated pallor and anemia requiring blood transfusions. Using next-generation sequencing, we discovered the responsible mutation in the proband but not in his parents; a heterozygous nucleotide variation of c.1000delA (p.1334Sfs*6) in *ANK1*. Thus hereditary spherocytosis was diagnosed.

**Conclusions:**

Genetic detection is an important means of discovering the cause of hemolytic anemia in neonates and infants where routine diagnostic tests are unrevealing. We found a novel de novo mutation, c.1000delA (p.1334Sfs*6) in *ANK1* that might account for other cases of HS in the Chinese population.

**Supplementary Information:**

The online version contains supplementary material available at 10.1186/s12920-021-00912-3.

## Background

Hereditary spherocytosis (HS) is a relatively common inherited disorder characterized by spherical-shaped red blood cells (RBCs) [1]. The erythrocyte membrane protein defects causing HS reduce erythrocyte stability and deformability, thereby increasing fragility producing hemolysis [2]. Approximately 75% of HS cases are autosomal dominant; the remaining 25% are either autosomal recessive or de novo mutations. Its prevalence is 1 in 2,000 in people of Northern European descent [3]. The clinical manifestations of HS in the neonatal age are typically hyperbilirubinemia followed by anemia. Owing to the commonness of neonatal jaundice, the diagnosis of HS can be missed in young infants, among patients whose parents are unaffected [4]. This particularly so when spherocytic erythrocytes are not prominent on the blood smear [5, 6]. Undiagnosed HS is one of the leading causes of kernicterus. Thus, early diagnosis may decrease long-term complications.

Mutations in five different genes are recognized to cause HS. These include Ankyrin (*ANK1*), α-spectrin (*SPTA1*), β-spectrin (*SPTB*), band 3 (*SLC4A1*), and protein 4.2 (*EPB42*). Worldwide, *ANK1* mutations are the most frequent causes of HS [7].

We diagnosed HS in an infant who had severe neonatal jaundice, where the cause was not identified until anemia had required two transfusions. We discovered a novel de novo* ANK1* mutation, c.1000delA (p.1334Sfs*6). This mutation could help expand the mutational genetic diagnosis of HS in jaundiced Chinese infants.

## Case presentation

### Methods

#### Next-generation sequencing

The GenCap custom enrichment kit with exon-tiling biotinylated capture probes (My Genostics China) is used to design a blood system panel for 816 genes (Additional file 1: Table S1) including ANK1, SPTA, SPTB, EPB41 and SLC4A1.

Peripheral blood samples were obtained from the neonate and his parents, and genomic DNA was purified using the QIAamp DNA Blood Mini Kit based on the manufacturer’s recommendations (Qiagen, Germany). A minimum of 3 µg genomic DNA were subjected to Illumina DNA library preparation according to manufacturer’s protocol (My Genostics, China). The enriched libraries (fragment distribution: 350–450 bp) were pooled and sequenced using Next Seq 500 (Illumina) to obtain 2 × 150 base paired-end reads.

Raw image files were processed using Bcl2Fastq software (2.18.0.12) to generate raw reads. Raw reads were trimmed for base call quality (quality score > 20) and adapter sequences using fastq-mcf tool.

Short reads were mapped to the human hg19 reference genome assembly using the Burrows-Wheeler aligner [6] (BWA 0.7.10).Aligned reads from BAM files were visualized using the Integrative Genomics Viewer (IGV 2.3) from the Broad Institute. Picard was used to remove duplicate reads and, Genome Analysis Tool Kit [8] (GATK 2.3.4) was used to obtain single nucleotide variations (SNVs) and short indels.

Filtered candidate variants listed in TSVC format files were annotated and interpreted by ANNOVAR [9], which contains an integrated functional annotation database including 1000 Genomes project, dbSNP and Clin Var were predicted by the PolyPhen-2 and SIFT tools were used to predict the functional effects of candidate variants on proteins [10]. Synonymous mutations were removed. Common variants with a minor allele frequency (MAF) > 5% in 1000 genomes Project, ExAc or ESP6500 were removed. The variants identified using this procedure were classified according to the American College of Medical Genetics and Genomics (ACMG) guidelines [11].

#### Variant confirmation using Sanger sequencing

The pathogenic and likely pathogenic variants were confirmed by Sanger DNA sequencing. Sanger validation primers for ANK1 variants were designed by Primer Premier. PCR samples were visualized on agarose gels and purified. Sequences were sequenced on an ABI PRISM 3730 genetic analyzer (ThermoFisher, USA) and compared with the human reference genome assembly (hg19).

### Case report and results

This male was born at 37 weeks 5 days gestation, weighing 2800 g. Apgar scores were 10 at 1 and 5 min. At ten hours he was transferred from the well baby unit to the neonatal department because of severe jaundice. His hemoglobin level was 138 g/L, reticulocyte count 4.3%, total bilirubin level 233.6 μmol/L and unconjugated bilirubin 196.7 μmol/L. Phototherapy was begun and an exchange transfusion performed in the first 24 h of life. Additional clinical information is provided in Table 1. The other routine laboratory tests were found to be negative.

**Table 1:** Hematological data on the patient

Address	DOI	Hb, g/dL	MCV, fL	MCH, pg	MCHC, g/dL	RBC count^a^	Ret, %
Reference		110–160	82–100	27–34	320–360	3.5–5.5	
A	9–29	138	101.8	35.1	345	3.93	4.3
A	10–1	106	98.7	35.6	363	2.98	
A	10–4	126	94.3	32.7	347	3.85	
B	11–29	69	80.2	28.4	354	2.43	4.6

DOI, date of investigation; A, women and children health institute; B, our hospital; Hb, hemoglobin; MCV, mean cell volume; MCH, mean cell hemoglobin; MCHC, mean cell hemoglobin concentration; RBC, red blood cell; Ret, reticulocyte ratio;^a^ × 10^12^/L

Spherocytes were not identified on the blood smear, but polychromatic erythrocytes and fragmented red cells were observed. The baby’s blood type was O (+). His mother’s blood type was A (+). Coombs test and G6PD screening were negative. Nine days later he was discharged home. About 30 days later, he was noted to be anemic again and a second blood transfusion was administered. He was admitted to our department at 60 days with pallor, anemia, splenomegaly (1 cm below the left costal margin) weighting 5.9 kg.

**Fig. 1:**
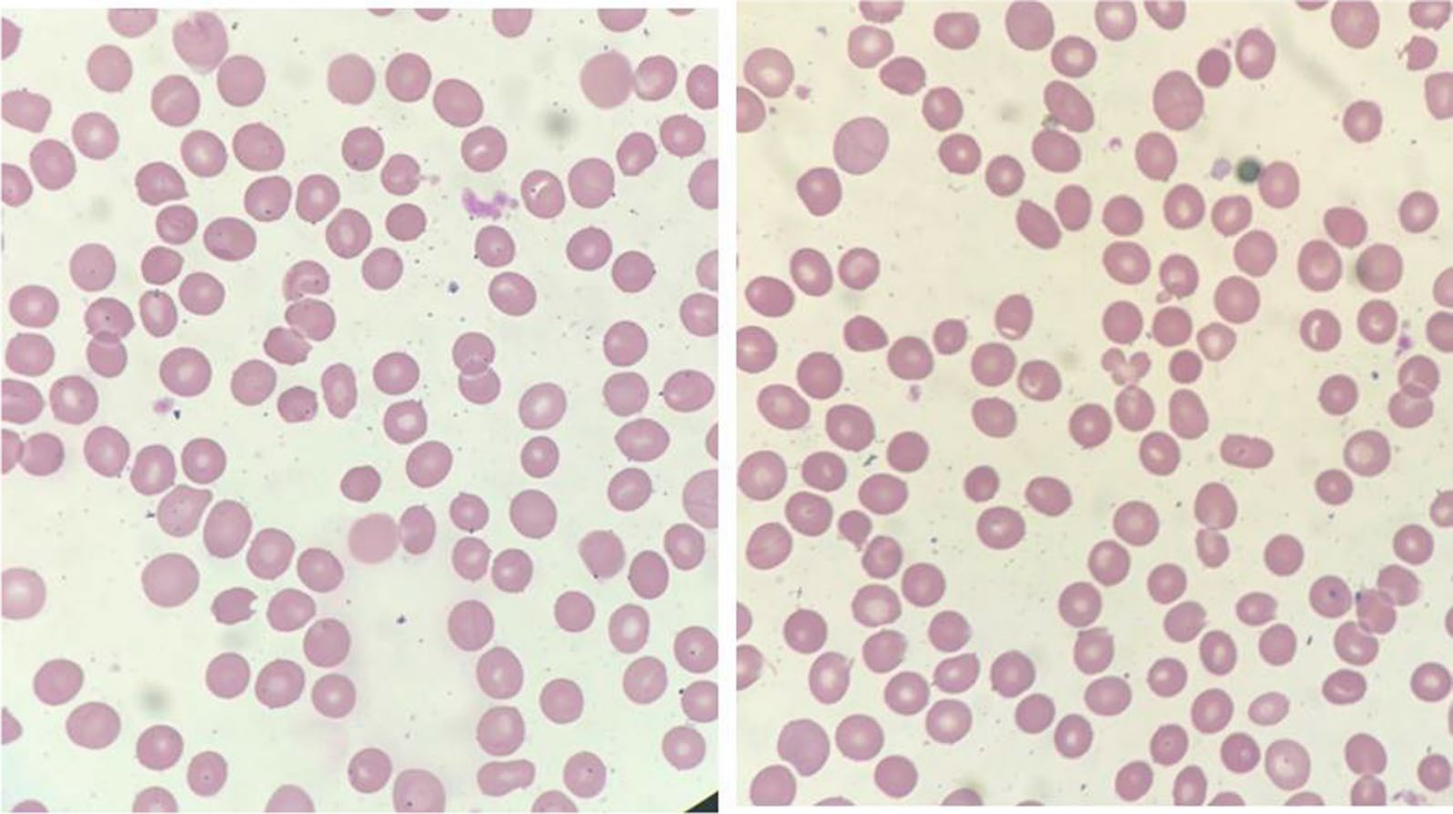
The peripheral blood smear

Laboratory studies at admission included: Hb 69 g/L, mean cell volume 80.2 fL, mean corpuscular hemoglobin concentration of 354.0 g/L, and reticulocyte count 4.6%. The peripheral blood smear did not show prominent spherocytes. There is less than 1% or none erythrocytes in each microscopic field which the process of making smear also can cause (Fig. 1). The presence of microcytic erythrocytes > 7.8% can be used for HS diagnosis [12].

Osmotic fragility test was abnormal, consistent with a diagnosis of HS. HbF was 5.2% by alkali-resistant Hb determination. The proband’s mother, father, and extended family members were well with no history of hyperbilirubinemia or anemia.

**Fig. 2:**
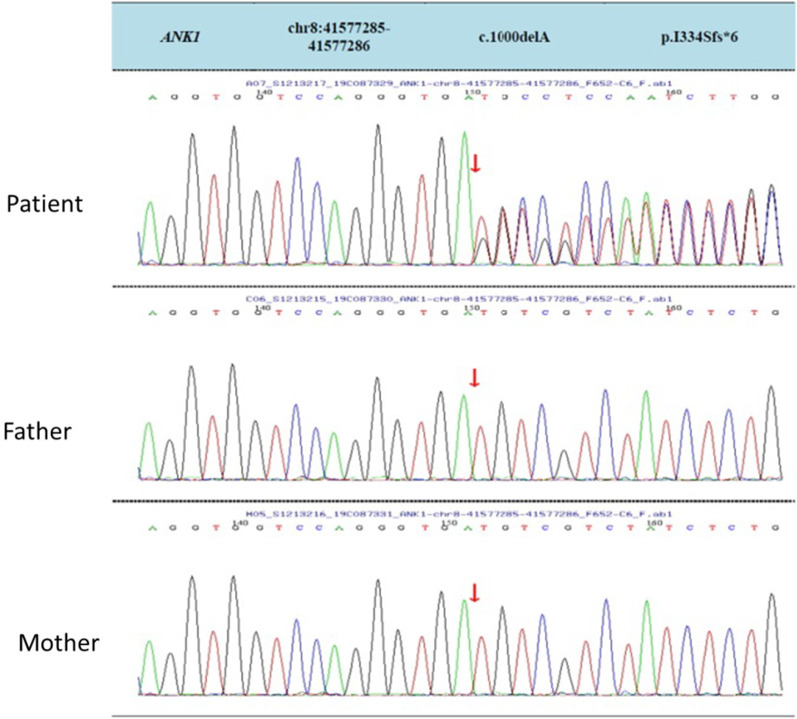
A novel de novo deletion mutation in the ANK1gene (NM_000037.3, c.1000delA) in the patient

We found a novel de novo deletion *ANK1* heterozygous mutation (c.1000delA, NM_000037.3) in the patient but in neither of his parents (Fig. 2). This mutation is not reported in the dbSNP database or literature. The ANK1 protein has 1881 amino acids and is composed of three structural domains: one membrane binding domain containing 24 Ankyrin repeats, one highly conserved spectrin-binding domain and a less conserved C-terminal regulatory domain. The ZU5A, ZU5B and UPA domains were identified in spectrin-binding domain and a “death” domain is found in the C-terminal regulatory domain [11].

**Fig. 3:**
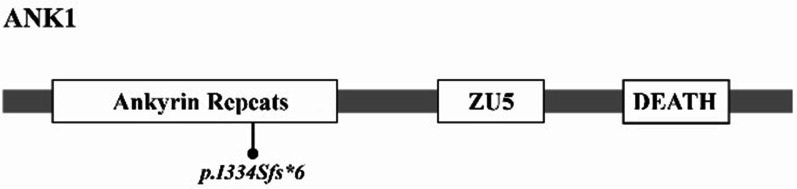
This frame shift mutation (c.1000delA) is located in the membrane binding domain of ANK1, occurring at 334 codon (p.I334Sfs*6)

This frame shift mutation (c.1000delA) is located in the membrane binding domain (Fig. 3), occurring at codon 334 codon and results in a premature termination codon (PTC) at the 340 codon (p.I334Sfs*6).This PTC will create a truncated protein without spectrin-binding domain and regulatory domain and result in loss function of ANK1 gene. Moreover according to the rule of Nonsense-mediated mRNA Decay (NMD), the mutated transcript with PTC may possibly be degraded by NMD pathway.

After being diagnosed with HS, the patient has received erythrocyte transfusions every month or two, with diminishing frequency. He is developing normally with no observed complications.

## Discussion and conclusions

HS is a congenital hemolytic disorder due to specific erythrocyte membrane protein abnormalities [13]. In the USA Kernicterus Registry, HS was the third most common genetic cause of hemolytic anemia-related kernicterus, behind G6PD deficiency and ABO hemolytic disease. However, the majority of kernicterus cases in the Registry were on the basis of “idiopathic” severe neonatal jaundice [14].

Diagnosing HS in a jaundiced neonate whose parents do not have this disorder is challenging [15–17]. This is illustrated in our case, where as a neonate he had significant jaundice, and then became anemic, yet he had no family history of HS and lacked abundant spherocytes on the blood film. It was not until two months had passed and two erythrocyte transfusions were needed that he was admitted with hemolytic anemia and NGS revealed the diagnosis.

The diagnostic criterion for HS is mean corpuscular hemoglobin concentration (MCHC) in guideline for the diagnosis and management of HS [2]. But its diagnostic sensitivity and specificity are not optimal [18]. Tao et al. [19] reported that mean sphered corpuscular volume (MSCV) had diagnostic value in HS. There are limiting clinical instruments that can be used to test MSCV [18]. Xu et al. [18] and Liao et al. [20] study showed that mean reticulocyte volume (MRV) is a general and specific new index for screening HS. But firstly the unification and standardization of the reference range need to be solved. Secondly there are no available in most of laboratory including our hospital. The EMA-binding test and other methods have complicated procedures and cannot be easily conducted in a typical laboratory. The new generation of ektacytometer is difficult to be applied in the clinical laboratory, mainly used in some research centers or specialized laboratories. The using of commercial intrusion NGS to detect exactly mutation gene is not expensive in China and may be easily speeded up and popular.

It is noteworthy that the *ANK1* mutation we found is both novel and de novo. De novo mutations are increasingly discovered in patients with severe early-onset diseases, such as neurodevelopmental disorders [21–25]. Our case supports the position that a more rigorous approach is needed to find underlying causes of “idiopathic” severe neonatal jaundice. In the Chinese population and in other populations as well, the specific mutation we identified, or other undiscovered mutations, could explain cases of severe “idiopathic” neonatal jaundice.

## Supplementary information

**Additional file 1: Table S1.** Analysis of the gene list.

## Data Availability

The DNA sequencing data generated during the current study are deposited in the NCBI Sequence Read Archive (SRA) repository under the accession number SRR13528516 (https://www.ncbi.nlm.nih.gov/sra/?term=SRR13528516). Public databases used in this study included human hg19 reference genome assembly (http://hgdownload.soe.ucsc.edu/goldenPath/hg19/bigZips/hg19.fa.gz), Human RefSeq Genome Annotation(https://www.ncbi.nlm.nih.gov/refseq/), ClinVar (https://www.ncbi.nlm.nih.gov/clinvar/), 1000genomes(http://www.1000genomes.org/), EXAC (http://exac.broadinstitute.org/), EVS (http://evs.gs.washington.edu/EVS), dbSNP (http://www.ncbi.nlm.nih.gov/projects/SNP/), HGMD (http://www.biobase-international.com/product/hgmd) and OMIM (https://www.omim.org/).

## Abbreviations

ANK1: Ankyrin
EPB42: Protein 4.2
G-6PD: Glucose-6 phosphate dehydrogenase
Hb: Hemoglobin
HS: Hereditary Spherocytosis
NGS: Next Generation sequencing
NMD: Nonsense-mediated mRNA Decay
MCV: Mean cell volume
MCHC: Mean Corpuscular Hemoglobin Concentration
MRV: Mean Reticulocyte Volume
M: Month
PTC: Premature termination codon
RBCs: Red blood cells
SPTA1: α-Spectrin
SPTB: β-Spectrin
SLC4A1: Band 3
